# Cranial ultrasound findings in late preterm infants and correlation with perinatal risk factors

**DOI:** 10.1186/s13052-015-0172-0

**Published:** 2015-09-24

**Authors:** Monica Fumagalli, Luca Antonio Ramenghi, Agnese De Carli, Laura Bassi, Pietro Farè, Francesca Dessimone, Silvia Pisoni, Ida Sirgiovanni, Michela Groppo, Alessandra Ometto, Dario Consonni, Fabio Triulzi, Fabio Mosca

**Affiliations:** NICU, Department of Clinical Sciences and Community Health, Fondazione IRCCS Cà Granda Ospedale Maggiore Policlinico, Università degli Studi di Milano, Milan, Italy; Neonatal Intensive Care Unit, Giannina Gaslini Paediatric Institute, IGG IRCCS, Genoa, Italy; Fondazione IRCCS Ca’ Granda Ospedale Maggiore Policlinico and Department of Occupational and Environmental Health, Unit of Epidemiology, Milan, Italy; Division of Neuroradiology, Fondazione IRCCS Cà Granda Ospedale Maggiore Policlinico Milano, Milan, Italy

**Keywords:** Late preterm infant, Cranial ultrasound, Brain injury, Perinatal risk factors

## Abstract

**Background:**

Late preterm infants are the most represented premature babies. They are exposed to a wide spectrum of brain lesions which are often clinically silent, supporting a possible role of cerebral ultrasound screening. Aim of the study is to describe the pattern of cranial ultrasound abnormalities in late preterm infants and to define the need for cranial ultrasound according to perinatal risk factors.

**Methods:**

A hospital-based cranial ultrasound screening was carried out by performing two scans (at 1 and 5 weeks). Unfavorable cranial ultrasound at 5 weeks was defined as either persistent periventricular hyperechogenicity or severe abnormalities.

**Results:**

One thousand one hundred seventy-two infants were included. Periventricular hyperechogenicity and severe abnormalities were observed in, respectively, 19.6 % and 1 % of late preterms at birth versus 1.8 % and 1.4 % at 5 weeks. Periventricular hyperechogenicity resolved in 91.3 %. At the univariate analysis gestational age (OR 0.5, 95 % CI 0.32-0.77), Apgar score <5 at 5’ (OR 15.3, 1.35-173) and comorbidities (OR 4.62, 2.39-8.98) predicted unfavorable ultrasound at 5 weeks. At the multivariate analysis the accuracy in predicting unfavorable ultrasound, estimated by combined gestational age/Apgar/comorbidities ROC curve, was fair (AUC 74.6) and increased to excellent (AUC 89.4) when ultrasound at birth was included.

**Conclusion:**

Gestational age and comorbitidies are the most important risk factors for detecting brain lesions. The combination of being born at 34 weeks and developing RDS represents the strongest indication to perform a cranial ultrasound. Differently from other studies, twin pregnancy doesn’t represent a risk factor.

## Background

Late preterm infants (LPIs) (defined as babies born between 34^+0^ and 36^+6^ weeks gestation) are the most represented premature infants (about 72 % of preterm births) in the developed countries, reaching 7–8 % of total live-births, and they account for the striking increase in premature birth which occurred in the last two decades [1].

The higher vulnerability of late preterm infants to develop diseases in the early neonatal period, compared to term babies, is well known. Mortality rate shows a 3-fold increase compared to term born controls and morbidity rates approximately double for each additional gestational week earlier than 38 weeks [1]. Late preterm infants have an increased risk of temperature instability, respiratory distress syndrome (RDS), excessive weight loss and dehydration requiring intravenous infusion, sepsis, hypoglycaemia and jaundice requiring phototherapy [2].

Brain vulnerability has also been documented in late preterm infants and neuromorbidity has been attributed to both extrinsic and intrinsic factors. The extrinsic vulnerability is related to detrimental effects of perinatal morbidities on the brain. On the other hand, intrinsic factors are related to the structural and molecular immaturity of the developing brain at specific gestational ages [3, 4]. Magnetic Resonance Imaging (MRI) studies have documented morphological maturational processes such as myelination, cortical folding and progressive involution of germinal matrix [5, 6], together with changes in specific functions like visual performances [7, 8].

The above-mentioned observations suggest a highly differentiated risk of neuromorbidities within the population of LPIs, being the younger babies born at 34 weeks at greater risk compared to the more mature LPIs born at 36 weeks.

In addition, they are exposed to a wider spectrum of brain lesions common to both most premature and more mature babies as they can develop not only germinal matrix-intraventricular hemorrhage (GMH-IVH) and cystic periventricular leukomalacia (cPVL) but also arterial/venous stroke [9], hypoxic-ischemic encephalopathy (HIE) [10], and those parenchymal injuries following hypoglycaemia. Most of these lesions are clinically subtle or silent in the neonatal period, remaining undiagnosed until later in childhood, and may contribute to explain the increased risk of impaired neurobehavioral outcome in LPs compared to term infants reported in the literature [11].

Early identification of LPs with brain abnormalities at cUS would allow early neurobehavioral intervention programs to improve long-term outcomes. However, considering the magnitude of the LP population, a universal cUS screening program would result in a heavy burden on caregivers, with increase in resource utilization and medical costs.

We therefore decided to perform a hospital-based cUS screening program aiming to describe the pattern of cUS abnormalities in the underinvestigated low-risk population of LPIs and to define the potential need for cUS according to perinatal risk factors.

## Methods

A cUS screening of LPIs was carried out between December 2010 and May 2013 in a single neonatal tertiary care center (Neonatal Intensive Care Unit, NICU, IRCCS Fondazione Ca’ Granda Ospedale Maggiore Policlinico, Milan, Italy). All inborn LPIs (34^+0^–36^+6^ weeks gestational age) were considered eligible.

Two cUS scans were performed: the 1^st^ within the first week of life (1^st^–7^th^ day of life) and the 2^nd^ at 5 weeks of life (28^th^–35^th^ day of life, corresponding to 39–41 weeks corrected age). Babies were excluded for parental refusal or when they missed one of the scheduled scans.

The following obstetrics and neonatal characteristics were collected: multiple birth and chorionicity, mode of delivery (vaginal delivery, vacuum extractor, elective or emergency caesarean section), gender, gestational age (GA), birth weight (BW), Apgar score at 1 and 5 min of life, ward at admission (postnatal ward or Neonatal Intensive Care Unit, NICU).

Comorbidities were classified as: transient tachypnea (TT, defined as tachypnea > 60 breaths/min shortly after delivery that usually resolves within 72 h and doesn’t require any assisted ventilation), respiratory distress syndrome (RDS, requiring either nasal continuous positive pressure, nCPAP, or invasive mechanical ventilation, MV; data on INtubation-SURfactant-Extubation procedure, INSURE, were also collected), hypoglycaemia (defined as at least 1 value of blood glucose < 30 mg/dl), HIE treated with hypothermia, congenital anomalies, necrotizing enterocolitis (NEC, requiring surgical treatment), sepsis (defined as increase in serum inflammatory markers and positive blood culture associated with clinical signs of infection). Morbidity at NICU admission was recorded and considered in the analysis.

CUS scans were performed by fellow paediatricians (P.F, M.G, S.P, F.D, A.D.C, I.S.) according to the clinical protocol of the unit and under the supervision of a team of neonatologists (L.A.R, M.F, L.B, A.O) with experience in neonatal brain imaging longer than 10 years. The fellows were previously trained in performing cUS by the same neonatologist (L.A.R.) for at least 6 months with specific agreement on difficult and ambiguous findings like congenital frontal pseudocysts and normal appearance of optical radiations. Investigators performing cUS were blinded to previous cUS scans.

Babies were scanned at bedside in supine position or in parents’ arms. Scans were performed with an ACUSON Sequoia® 512 US machine using a convex transducer with frequency of 7.5 MHz.

CUS report included the description of: ventricular system, midline structures, parenchymal echogenicity, posterior fossa structures. Ventricular dilatation was estimated according to the measurement of anterior horn width [12] and of the thalamo-occipital distance [13].

The parenchymal echogenicity in the periventricular areas was defined as periventricular hyperechogenicity (PHE) when isoechogenic/hyperechogenic to the choroid plexus.

CUS findings were classified as: 1. normal; 2. mild abnormalities: asymmetric lateral ventricles, mild dilatation of the occipital horns (thalamo-occipital distance <95 percentiles), cysts of the chorioid plexus, frontal, temporal and caudothalamic pseudocysts, lenticulostriate vasculopathy; 3. PHE; 4. severe abnormalities: GHM-IVH, defined according to the Papile’s criteria [14], cPVL, venous/arterial stroke and malformations.

### Ethics, consent and permissions

All procedures performed in the study were in accordance with the ethical standards of the institutional and national research committee and with the 1964 Helsinki declaration and its later amendments or comparable ethical standards. Informed consent was obtained from the parents of participants included in the study. Review board of Fondazione IRCCS Ca' Granda Ospedale Maggiore Policlinico di Milano approved the study.

### Statistical analysis

Agreement between cUS scans over time was examined using Cohen’s kappa statistics. We fitted univariate and multiple logistic regression models to calculate the odds ratio (OR) and 95 % confidence interval (CI) of having PHE or severe abnormalities at 5 weeks. To evaluate the usefulness of cUS when added to other selected clinical variables, after the logistic models we estimated the receiver operating characteristic (ROC) curves and their area under the curve (AUC). Statistical analyses were performed with Stata 12.

## Results

1588 LPIs were eligible, 1172 completed the protocol and were included (208 babies were not recruited at birth and 208 missed the 2^nd^ scan).

The characteristics of the study population and early neonatal morbidities are shown in Table 1. Early neonatal morbidities were reported as follows: transient tachypnea in 25 babies (2.1 %), RDS requiring nCPAP in 102 (8.7 %), INSURE in 19 (1.6 %), mechanical ventilation in 45 (3.8 %), hypoglycaemia in 22 (1.8 %), HIE treated with hypothermia in 3 (0.2), congenital malformation at birth in 34 (3 %), NEC in 3 (0.2 %). No sepsis was observed.

**Table 1:** Characteristics of the study population

Variables		*N* (%)
Sex (*N* = 1172)	Male	608 (51.9)
	Female	564 (48.1)
GA (*N* = 1172)	34 weeks	227 (19.4)
	35 weeks	355 (30.4)
	36 weeks	590 (50.3)
Twin birth	Singleton	641 (54.7)
	BC-BA	426 (36.3)
	MC-BA	82 (7)
	MC-MA	2 (0.2)
	Trigeminal	21 (1.8)
Apgar (*N* = 1172)	≤5 at 1’	29 (2.5)
	≤5 at 5’	3 (0.2)
Mode of delivery (*N* = 1172)	Vaginal delivery	258 (22)
	Vacuum extractor	21 (1.8)
	Elective CS	416 (36)
	Emergency CS	477 (40.7)
Ward at admission (*N* = 1172)	Postnatal ward	913 (78)
	NICU	259 (22)
Birthweight	Mean (g)	SD (g)
34 weeks (*N* = 227)	2167	403,2
35 weeks (*N* = 355)	2375	408,2
36 weeks (*N* = 590)	2556	401,6

*GA* gestational age, *BC* bichorionic, *BA* biamniotic, *MC* monochorionic , *MA* monoamniotic, *CS* cesarean section, *NICU* Neonatal Intensive Care Unit, *g* grams, *SD* standard deviation

Changes in the incidence of cUS findings between 1^st^ and 2^nd^ cUS are shown in Table 2. PHE and severe cUS abnormalities were observed in, respectively, 19.6 % and 1 % of LPIs at birth versus 1.8 % and 1.4 % at 5 weeks. Agreement between cUS scans over time was fair (weighted Kappa = 0.28), mainly due to the transient nature of PHE (Table 2). Two hundred ten (91.3 %) out of 230 babies with PHE at the 1^st^ scan had either normal or mild abnormal findings at the 2^nd^ cUS scan. PHE persisted in twenty-one infants and these babies underwent MRI scan: multiple punctuate lesions were observed in 2 babies, not-cystic PVL (white matter loss and squared lateral ventricles) in 3 infants.

**Table 2:** Changes in the incidence of cUS findings and agreement between 1^st^ and 2^nd^ cUS scan (N;%)

	ᅟ

Grey areas represent discordance between 1^st^ and 2^nd^ scan; dark grey areas indicate false negative and light grey ones false positive

Severe cUS abnormalities were observed in 17 babies (1.4 %) at 5 weeks: 2 cPVL, 4 arterial stroke, 2 venous infarction, 3 malformations (Blake’s pouch, agenesis of the corpus callosum, arachnoid cyst), 4 GMH-IVH (2 IVH grade 1, 2 IVH grade 2), 1 central grey matter and white matter lesions consistent with HIE and 1case of diffuse periventricular and subcortical patchy hyperechogenicity (due to congenital Cytomegalovirus infection). In 4/17 babies the first cUS did not show the lesion which was detected at the 2^nd^ scan (2 arterial stroke, 1 small venous infarction and 1 IVH grade 1). In the two infants later developing cPVL, the 1^st^ cUS presented PHE indistinguishable from the other ones .

All severe cUS abnormalities were confirmed by MRI.

The univariate regression (Table 3) was used to investigate the association between perinatal risk factors and abnormal cUS at 5 weeks, defined as either persistent PHE or severe abnormalities. Younger GA (categorized by week of GA, p 0.002), Apgar score ≤ 5 at 1 and 5 min (*p* =0.006 and *p* = 0.028 respectively) and the presence of comorbidities (p < 0.001), were more common in babies with abnormal cUS at 5 weeks. The risk of abnormal cUS (PHE/severe abnormalities) was inversely related to GA and doubled for every week gestation moving from 36 to 34 (34 weeks OR 1 -reference; 35 weeks OR 0.54, 95 % CI 0.22-1.25; 36 weeks OR 0.25, 95 % CI 0.99-0.6). RDS requiring either invasive or non-invasive ventilatory support was the most common neonatal morbidity and was significantly associated with the occurrence of abnormal cUS findings. Hypoglycaemia was also associated with cUS findings; however, due to the small number of observations (2 babies) this result deserves further confirmation.

**Table 3:** Univariate logistic regression for variables

		*PHE + severe cUS at 5 weeks*			
		Number	*OR (95 % C.I.)*	*p*	*ROC*
Sex	Male	21	1 (reference)		0.52
	Female	17	0.87 (0.44; 1.70)	0.69	
GA	34-36	38	0.50 (0.32; 0.77)	0.002	0.65
Twins	Singleton	25	1 (reference)		0.06
	MC-BA	1	0.30 (0.04; 2.25)	0.24	
	BC-BA	9	0.53 (0.23; 1.21)	0.13	
	Trigeminal	3	4.10 (0.47; 35.4)	0.2	
Apgar 1’	>5	33	1 (reference)		0.54
	≤5	5	4.14 (1.51; 11.3)	0.006	
Apgar 5’	>5	37	1 (reference)		0.51
	≤5	1	15.3 (1.35; 173)	0.03	
Delivery	Vaginal	9	1 (reference)		0.53
	Vacuum	1	1.37 (0.18; 10.7)	0.76	
	Elective CS	11	0.74 (0.27; 2.09)	0.58	
	Emergency CS	16	0.96 (0.42; 2.21)	0.92	
Comorbidity	No (all)	17	1 (reference)		0.67
	Yes (all)	21	4.62 (2.39; 8.98)	<0.001	
	TT	0	-		0.68
	nCPAP	10	5.52 (2.52; 12.11)	<0.001	
	INSURE	0	-		
	MV	7	9.18 (3.5; 24.1)	<0.001	
	Hypoglycaemia	2	5.25 (1.1; 25.0)	0.04	
	Malformation	2	3.28 (0.71; 15.1)	0.13	
	NEC	0	-		
1st cUS	Normal	6	1 (reference)		0.84
	Mild	1	1.40 (0.17; 11.8)	0.76	
	PHE	20	13.1 (9.97; 34.4)	<0.001	
	Severe	11	1511 (167; 13643)	<0.001	

*BC* bichorionic, *BA* biamniotic, *MC* monochorionic, *CS* cesarean section, *TT* transient tachypnea, *INSURE* Intubation - SURfactant- Extubation, *MV* mechanical ventilation, *NEC* necrotizing enterocolitis, *cUS* cranial ultrasound, *PHE* periventricular hyperechogenicity

**Fig. 1:**
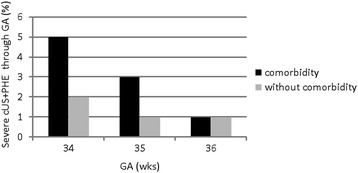
Severe cerebral ultrasound (cUS) abnormalities and periventricular hyperechogenicity (PHE) at 5 weeks according to gestational age (GA) and the occurrence of comorbidities

Figure 1 shows the distribution of severe cUS abnormalities and PHE at 5 weeks according to GA at birth and the occurrence of comorbidities.

**Fig. 2:**
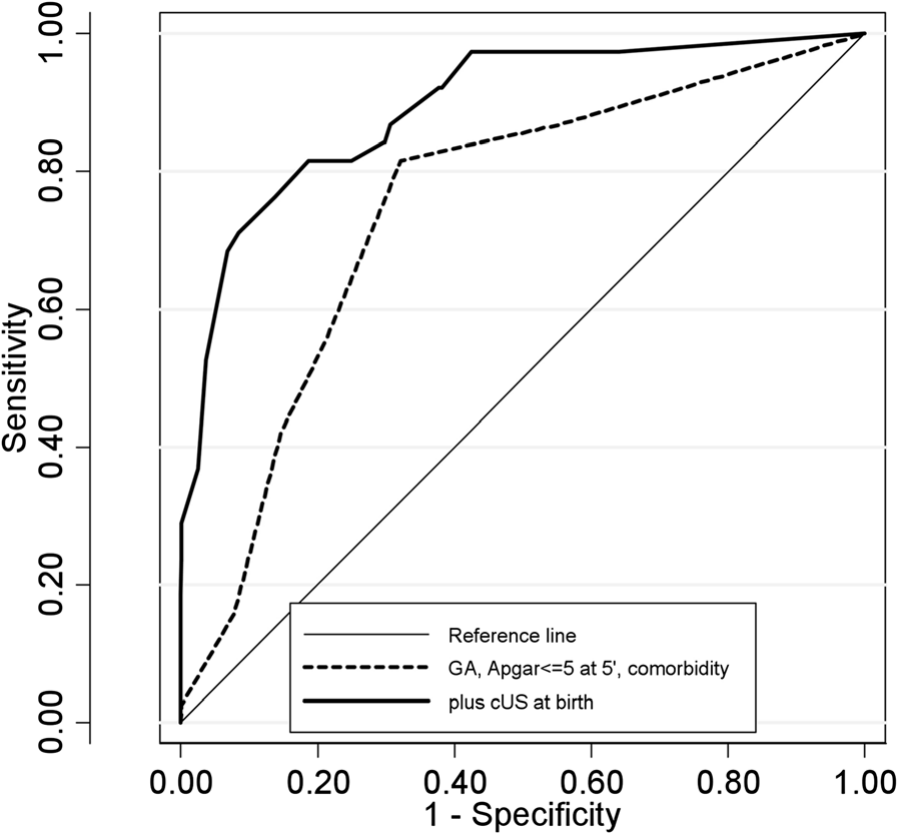
ROC curves for variables. The thin continuous line represents the reference model; the dashed line represents the model with gestational age (GA), Apgar Score ≤ 5 at 5’ and comorbidity and the bold continuous line the model with GA, Apgar Score ≤ 5 at 5’, comorbidity and cerebral ultrasound at birth

At the multivariate analysis (Table 4) the accuracy in predicting unfavorable cUS at 5 weeks, estimated by combined GA/Apgar at 5’/comorbidities ROC curve, was fair (AUC 74.6) but increased to excellent (AUC 89.4) when cUS findings at birth (classified as normal, mild or severe abnormal and PHE) were included (Fig. 2).

**Table 4:** Multiple logistic regression

		PHE + severe cUS at 5 weeks		
		Number	OR (95 % C.I.)	*p*
GA	34-36	38	0.60 (0.33; 1.10)	0.09
Apgar 5’	>5	37	1 (reference)	
	≤5	1	1.64 (0.27; 10)	0.59
Comorbidity	No (all)	17	1 (reference)	
	Yes (all)	21	2.16 (0.88; 5.31)	0.09
cUS at birth	Normal	6	1 (reference)	
	Mild	1	1.23 (0.14; 10.5)	0.84
	PHE	20	10.2 (3.9; 26.5)	<0.001
	Severe	11	1150 (115; 11450)	<0.001
Area under ROC curve: 0.89				

*GA* gestational age, *cUS* cranial ultrasound, *PHE* periventricular hyperechogenicity

## Discussion

Our data support the hypothesis that gestational age at birth and the occurrence of neonatal comorbidities are the most important risk factors for detecting brain lesions in the late preterm population.

The combination of being born at 34 weeks gestation and the occurrence of RDS represents the strongest indication to perform a cUS scan. Being born a week later, at 35 weeks gestation, reduces the absolute risk of developing severe brain lesions but it persists in case of comorbidities. This does not seem to be the case, any more, at 36 weeks when prematurity and comorbidities play an equivalent role.

The impact of timing of cUS on detecting brain lesions was investigated. At the 1^st^ cUS we missed 4 out of 17 severe brain lesions detected at 5 weeks: 2 arterial stroke, 1 venous infarction and GMH- IVH grade 1 in one case. GMH-IVH mostly occurs during the first 72 h of age and a very early 1^st^ scan (within the defined interval time 1^st^–7^th^ day of life) may have missed it. The problem of undetected GMH-IVH may be further compounded by recent evidence showing that cUS sensitivity in detecting grade 1–2 GMH- IVH is surprisingly low, compared to MRI [15].

GMH-IVH is a rare although unusual event in late prematurity. In addition, also minor form occurring in VLBW babies are associated with impaired neurological outcome as very recent and robust studies demonstrated [16]. Thus, we included also grade I-II GMH- IVH in the unfavorable cUS. These negative effects are consistent with germinal matrix destruction and loss of astrocytic precursor cells [17] or with periventricular white matter inflammation due to astrocytes activation triggered by the long persistence of haemosiderin along the ependyma [18].

Difficulties in performing an early diagnosis tend to occur also with arterial stroke, a brain lesion developing within the first week, rarely beyond day 3 of life, but becoming more obvious at cUS over the following few days.

The fair agreement between cUS scans over time (1^st^ versus 2^nd^ scan) was mainly related to the transient nature of PHE. PHE was the most represented cUS abnormality in LPIs at birth although it disappeared within 5 weeks in 91 % of infants. It is a common finding in very preterm infants in the first week of life and it is more pronounced with declining GA. It can be either pathological (pre-cystic phase of cPVL) or transient (related to increased water content) and not resulting in a definite lesion [19]. Transient PHE, still detectable beyond the first week of life, can be classified as PVL I according to de Vries [19] and the duration of flaring is directly related to the severity of brain injury. However, the significance and the evolution of PHE in the more mature LPIs are still unclear. For this reason, we decided to scan babies for a second time at 5 weeks of age in order to detect both prolonged pathological flaring (defined by a duration of 14 days or more according to Dammann and Leviton) [20] and also those cavitations developing so late after birth. Our choice to perform a second, so late, scan is supported by the evidence that cPVL still occurs among early preterm infants (28–35 weeks) despite the dramatic decrease in its incidence at the youngest gestational ages (24–27 weeks) [21].

All babies with prolonged PHE at 5 weeks underwent MRI between 40 and 44 weeks corrected age disclosing white matter abnormalities in 23.8 % of infants: white matter loss like proper PVL in 3 out of 5 infants and punctate lesions in 2 babies [21] but there were no cases of proper cPVL. All these 5 babies presented with severe morbidities (RDS) at birth requiring NICU admission. In the remaining 76.2 % of babies with prolonged PHE conventional MRI did not reveal any white matter abnormalities. Several considerations support the low correlation we observed between cUS and MRI in diagnosing white matter abnormalities in this specific population, compared to previous published studies. Most of the studies comparing cUS and MRI on detection of white matter abnormalities [22–24] have been performed in very preterm infants (<32 weeks gestation) while changes in periventricular flaring after birth and correlation with MRI in the more mature LP population are still underinvestigated. Moreover, in previous studies, unlike the present one, diffuse excessive high-signal intensity on T2-weighted MR images (the so called DEHSI) has been considered part of the spectrum of white matter injury. However, according to recent evidence DEHSI should be considered a developmental phenomenon related to prematurity rather than a pathological finding itself and this theory is well supported by the lack of correlation of this common MR finding in preterm infants at term with adverse long-term neurobehavioral outcome [25, 26].

In preterm infants, RDS requiring mechanical ventilation has been associated with fluctuation of cerebral blood flow in the first days of life [27] and increased risk of brain injury, mainly IVH. Cerebrovascular autoregulation and reactivity play a role in brain injury in premature babies and mechanical ventilation may interfere with these physiological mechanisms by affecting systemic haemodynamics and modulating arterial carbon dioxide tension [28, 29].

The causative association between comorbidities and severe cUS abnormalities deserves further analysis as they seem to act as a second “hit” triggering or aggravating pathological processes in the developing brain of premature babies and affecting both the white matter and the involuting structures, such as the germinal matrix.

Although we could not estimate the incidence of brain lesions in the LP population, as the study was not powered to do it, we confirmed previous observations suggesting that LPIs can develop a wide spectrum of cerebral lesions common to both most premature and more mature babies.

The univariate analysis did not support the possible role of twin birth as risk factor for brain lesions in LPIs. Twin pregnancies carry a higher risk of neonatal death, cerebral palsy and intrauterine death [30]. However, increased neonatal morbidity appears to be related to prematurity rather than to twin birth itself although monochorionicity seems to play a detrimental role, in particular when complicated by twin-to-twin transfusion syndrome. This study did not confirm these observations: 45 % of the LPIs enrolled were born from twin pregnancies but this condition did not represent a risk factor for brain lesions, not even for babies born from monochorionic pregnancies.

## Conclusions

At the lower gestational age, within the late preterm period, the intrinsic vulnerability of the developing brain increases the risk of developing brain lesions in particular when extrinsic factors, such as comorbidities, coexist. The indication to perform a cranial ultrasound scan in a late preterm infant should be modulated according to gestational age and the severity of the postnatal course, in particular the occurrence of respiratory distress syndrome.

## Abbreviations

AUC: Area under curve
BW: Birth weight
CI: Confidence interval
cPVL: Cystic periventricular leukomalacia
cUS: Cranial ultrasound
GA: Gestational age
GMH-IVH: Germinal matrix hemorrhage-intraventricular hemorrhage
HIE: Hypoxic-ischemic encephalopathy
INSURE: INtubation-SURFactant-Extubation
LPIs: Late preterm infants
MRI: Magnetic resonance imaging
MV: Mechanical ventilation
nCPAP: Nasal continuous positive pressure
NEC: Necrotizing enterocolitis
OR: Odds ratio
PHE: Persistent hyperecogenicity
RDS: Respiratory distress syndrome
ROC: Receiver operating characteristic
TT: Transient tachypnea
